# Fluid Extract of Gelsemium in Tetanus

**Published:** 1896-09

**Authors:** W. G. Hollingsworth

**Affiliations:** Utica, N. Y.


### FLUID EXTRACT OF GELSEMIUM IN TETANUS.

By W. G. HOLLINGSWORTH, D.V.S.,
UTICA, N. Y.

I DESIRE to report my success with the above remedy in tetanus.

My first case, a bay gelding, sixteen and one-half hands high, used for coach purposes, owned by President Walcott, of the New York Mills Co. On visiting the animal I found a well-developed case, arising through a punctured wound of the foot, which I treated antiseptically. Placed him under half-ounce doses of the fluid extract every half hour, per rectum, as there was so much trismus I was unable to give by mouth. This treatment was continued for four days, when there was a change: muscles commenced to relax, ears move, animal could place head to bottom of manger. I used slings, which I think are to be advised in all cases. After the fourth day medicine was given by the mouth in two-drachm doses, gradually lessening in frequency as the animal improved. This animal was under treatment for three weeks. Is now as limber and supple as he could be. He received four pints in all of the gelsemium.

The second case, a mare, was much worse, with almost continual spasms. The same treatment was prescribed. I used slings. The spasms stopped on the second day. On the third day she could eat grass, and from this time continued to improve daily, and is now working.

In those cases where I have not used slings the animals got down, and that generally settled them. Two other cases treated as above made good recoveries. I find efforts to give medicine by the mouth difficult, and cause more or less annoyance to the animal. My success with this remedy has suggested reporting the same.

